# Sub‐3 nm Intermetallic Ordered Pt_3_In Clusters for Oxygen Reduction Reaction

**DOI:** 10.1002/advs.201901279

**Published:** 2019-11-18

**Authors:** Qi Wang, Zhi Liang Zhao, Zhe Zhang, Tianli Feng, Ruyi Zhong, Hu Xu, Sokrates T. Pantelides, Meng Gu

**Affiliations:** ^1^ Department of Materials Science and Engineering Southern University of Science and Technology Shenzhen 518055 China; ^2^ Department of Materials Science and Engineering University of Science and Technology of China Hefei 230026 P. R. China; ^3^ Department of Physics Southern University of Science and Technology Shenzhen 518055 China; ^4^ Department of Physics and Astronomy and Department of Electrical Engineering and Computer Science Vanderbilt University Nashville TN 37235 USA

**Keywords:** intermetallic ordered Pt_3_In, large‐scale, oxygen reduction reaction, sub‐3 nm clusters, theoretically minimum size

## Abstract

Industrial applications of Pt‐based oxygen‐reduction‐reaction (ORR) catalysts are limited by high cost and low stability. Here, facile large‐scale synthesis of sub‐3‐nm ordered Pt_3_In clusters on commercial carbon black as ORR catalyst that alleviates both these shortcomings is reported. As‐prepared Pt_3_In/C exhibits a mass activity of 0.71 mA mg^−1^ and a specific area activity of 0.91 mA cm^−2^ at 0.9 V vs reversible hydrogen electrode, which are 4.1 and 2.7 times the corresponding values of commercial Pt/C catalysts. The as‐prepared ordered Pt_3_In/C catalyst is also remarkably stable with negligible activity and structural decay after 20 000 accelerated electrochemical durability cycles, due to its ordered structure. Density‐functional‐theory calculations demonstrate that ordered‐Pt_3_In is more energetically favorable for ORR than the commercial Pt/C catalysts because ∆*G*_O_ is closer to the peak of the volcano plot after ordered incorporation of indium atoms.

## Graphical abstract

An ordered Pt_3_In/C catalyst with a minimum ordered size of 1 nm and an average size of 1.9 nm is synthesized with a facile method that can be easily scaled up. The product exhibits a high mass activity that is 4.1 times the corresponding value of the Pt/C catalyst, but costs only 1/4 of its price.

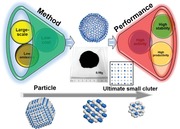

## 1 Introduction

Concerning environmental pollution and the energy crisis caused by the depletion of traditional fossil resources, great efforts have been put into the development of renewable energy.^1^, ^2^ Fuel cells have been considered as green‐energy devices for electric vehicles and portable power due to their high energy‐conversion efficiency, eco‐friendly products, and reliability.^3^ However, the sluggish kinetics of ORR on the cathode limit their applications.^4^ Currently, the noble metal platinum (Pt) is the most commonly used catalyst for fuel cells. Nevertheless, the high cost, poor durability, and limited reserves of Pt catalysts have been the major obstacles for the commercialization of fuel cells.

Previous works have found that the surface atomic structure and composition of catalysts play critical roles in the electrocatalytic performance of Pt‐based catalysts.^5^, ^6^, ^7^ Accordingly, nanoalloys with various atomic arrangement style including controlled composition or shape, core–shell structure, heterostructure, hollow structure and ordered structure have been designed and proved to be effective ways to modify the catalytic performance of Pt‐based catalyst.^8^, ^9^, ^10^, ^11^, ^12^ Furthermore, well‐designed alloyed particles with Pt‐enriched surfaces should possess a suitable electronic structure for ORR and thus meet both high performance and lower cost.^13^ Low‐coordination metal atoms often function as the catalytically active sites, while the specific activity per metal atom usually increases with decreasing size of the metal particles.^14^, ^15^ Additionally, smaller size also indicates higher dispersion of Pt atoms and higher utilization efficiency.^16^ However, the surface free energy of metals increases significantly with decreasing particle size, promoting aggregation of small clusters.^17^

Monodispersed superfine alloyed clusters have many advantages, such as high Pt‐utilization efficiency and synergistic effects arising from neighboring metal atoms.^18^ More importantly, by forming ordered intermetallic PtM nanocrystals with some metal M, the higher mixing enthalpy and stronger atomic interactions between Pt and M atoms would make PtM highly stable under electrochemical tests in both acidic and alkaline solutions.^19^, ^20^, ^21^, ^22^ However, in general, forming structurally ordered intermetallic phases requires high‐temperature annealing (>500 °C), while such high temperatures are detrimental to maintaining the ultrasmall nanocluster size. Though some strategies like protection by surfactants, salt encapsulation, and oxide or polymer coatings offer the promise to realize ordered nanoalloys with certain size,^23^, ^24^ direct and large‐scale fabrication of cluster‐level ordered nanostructures has not been reported.

In this work, novel sub‐3‐nm ordered Pt_3_In clusters have been synthesized via a facile surfactant‐free method for the first time. The as‐prepared Pt_3_In clusters exhibit a mass activity of 0.71 mA mg^−1^_Pt_ and a specific activity of 0.91 mA cm^−2^_Pt_. The intermetallic Pt_3_In/C catalysts can endure at least 20 000 accelerated electrochemical durability test cycles, with negligible activity decay and structural depletion. To explore the ORR mechanism, density functional theory (DFT) calculations were carried out and the results show that, after the ordered incorporation of In atoms into Pt lattice, the ∆*G*_O_ of ordered Pt_3_In clusters is closer to the peak of the volcano plot. In addition, the cost of as‐synthesized ordered Pt_3_In catalyst is about 25% the price of commercial Pt/C with the same Pt content. The substantial activity, excellent stability, and low cost of the large‐scale synthesis protocol reported here have the potential to meet the long‐pursued goal of commercial catalysts for fuel cells.

## 2 Result and Discussion

**Figure 1:**
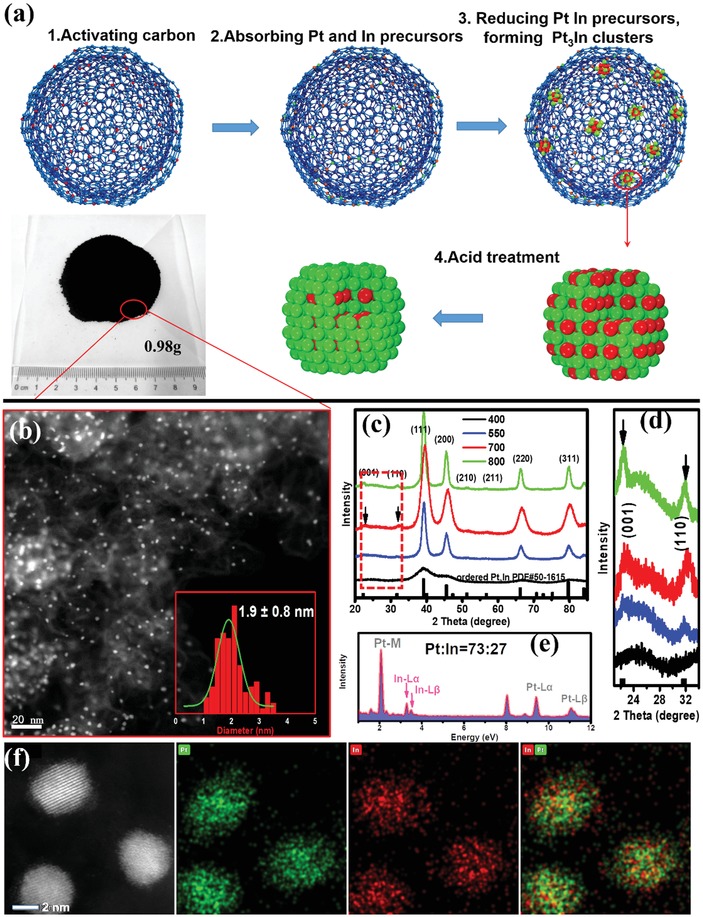
a) Schematic illustration of the synthesis procedure of ordered Pt_3_In/C‐T700 catalysts. Inset shows a gram‐scale product in a one‐batch synthesis. b) Representative STEM image of as prepared catalysts. Inset shows a size distribution histogram with an average size of 1.9 nm. c) XRD patterns of as‐synthesized catalysts under different temperatures (400, 550, 700, 800) for 2 h. d) A magnified part in the red square of (c) shows the superlattice diffraction peaks. e) EDX result of the as‐prepared catalyst. f) Elemental mappings of ordered Pt_3_In particles.

The schematic graphic is shown in **Figure** **1**a illustrating the preparation process of intermetallic Pt_3_In/C. Due to the presence of defects and oxygen‐containing functional groups on the surface of HNO_3_‐treated carbon black, the carbon black support possesses a high adsorption capacity for metal cations. The In and Pt precursors were then introduced and sonicated with the activated carbon black. After drying the suspension by vacuum rotary evaporation, the powder was heated in a tube furnace under H_2_ flow at different temperatures for 2 h. Excess surface indium atoms were then removed by washing with HClO_4_ and finally gram‐scale catalysts were synthesized. Figure 1b shows a typical scanning transmission electron microscopy (STEM) image of Pt_3_In/C‐T700, indicating the ultrafine and evenly distributed cluster. Higher and lower STEM images in Figure S5 of the Supporting Information give an overall understanding of the cluster.

The structure difference between Pt_3_In samples treated at different temperature was analyzed by X‐ray diffraction (XRD). As illustrated in Figure 1c,d, the Pt_3_In particles show broad peaks at near 39.13°, 45.55°, 66.23°, and 79.63°, which is consistent with XRD patterns for a disordered alloy of a face center cubic (FCC) structure. The diffraction peaks for Pt_3_In are shifted to lower 2θ values compared with those for Pt/C‐JM, which can be clearly observed in Figure 1c, indicating that In atoms are incorporated into the Pt FCC structure to form an alloy phase with expanded lattice. With increasing treating temperature, the peaks become sharper, indicating growth of particle size. Notably, the intensity of ordered (100) and (110) diffraction peaks become stronger with the increasing temperature, demonstrating the increased ordered degree with higher annealing temperature (Figure 1d). However, the relative intensity of these two peaks with (111) peaks in sample Pt_3_In/C‐T700 and Pt_3_In/C‐T800 are almost the same, implying that the disordered to ordered transformation process completed at 700 °C for 2 h and higher temperature did not further improve the order degree.

**Figure 2:**
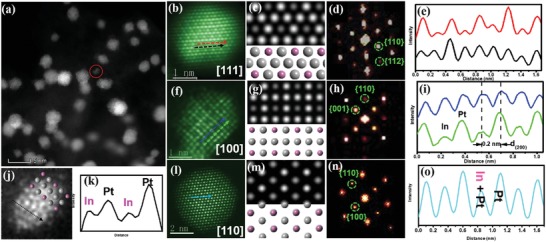
a) STEM image of Pt3In/C‐T700 samples, showing highly ordered particles. STEM images of individual particle along b) [111], f) [100], and i) [110] zone axes, respectively. c,g,m) Corresponding simulated STEM images and atomic models. d,h,n) FFT of (b), (f), and (i). e,i,o) Intensity profile of (b), (f), and (i). j) STEM image of ultrafine ordered cluster and k) corresponding intensity profile.

In order to understand the ordering transformation of ordered Pt_3_In bimetallic clusters, we probed the structure of ordered Pt_3_In/C‐T700 using various methods. Elemental analysis mapping of the distribution of In and Pt showed that both elements were uniformly distributed in a single cluster for both ordered (Figure 1f) and disordered clusters. For the insights down to the atomic level, aberration‐corrected high‐angle annular dark‐field (HAADF)‐STEM images of ordered Pt_3_In in a relatively low magnification are shown in **Figure** **2**a. Ordered structure was observed in almost every particle presented in Figure 2a, indicating high ordering degree. An atomically resolved STEM image in Figure 2b shows the atomic arrangement view along the [111] direction, judging from the corresponding FFT pattern (Figure 2d). The atomic model and simulated STEM image along this zone axis are shown in Figure 2c, which is consistent with the experimental results. From the images, a periodic oscillation of intensity was observed (Figure 2e), which can be attributed to the Z‐contrast differences between Pt and In in an ordered lattice. A column of Pt atoms appears much brighter than that of In atoms in an ordered structure. By distinguishing elements from their contrast and measuring the distance between the columns, certain unit cell orientations of the L1_2_ structure were identified. Additional atomic‐structure data along the [100] and [110] directions (Figure 2f,l), corresponding models and STEM simulations (Figure 2g,m) and FFT (Figure 2h,n) further confirm the particle to be an intermetallic structure. Besides, a Pt‐enriched surface was found in the [001] zone, which could act as stable shell during cycling. Figure 2i shows an ordered cluster with diameter 1 nm (4 atomic layers), which is the smallest ordered structure ever reported in ORR catalysts. In an attempt to understand the thermal stability of these ultrasmall ordered clusters after 700 °C treatment, ab initio molecular dynamics simulations were carried out. As shown in Figure S7 of the Supporting Information, we see that the particles with size larger than 1.2 nm are stable at 700 °C. However, ordered clusters that are smaller than 1.2 nm collapse spontaneously. Combining with the experimental results, it is plausible that the smallest ordered alloy structure is obtained through the synthesis protocol.

**Figure 3:**
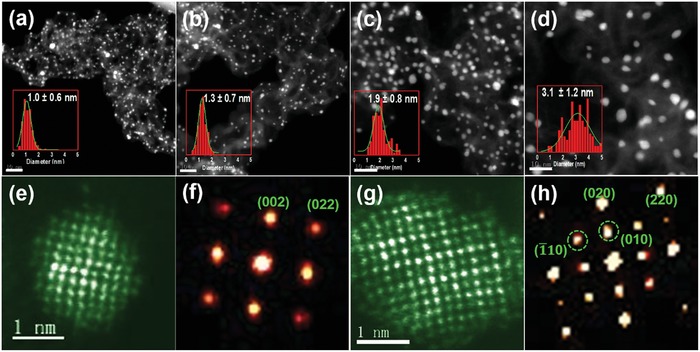
a–d) HAADF‐STEM images of Pt_3_In/C‐T400, Pt_3_In/C‐T550, Pt_3_In/C‐T700, and Pt_3_In/C‐T800. Insets are the corresponding size distribution histograms (scale bar is 10 nm). e) STEM image of an individual particle of Pt_3_In/C‐T400 and f) corresponding FFT. g) STEM image of an individual particle of Pt_3_In/C‐T700 and h) corresponding FFT. Dashed green circles in panel h indicate the (110) and (001) diffraction spots corresponding to ordered Pt_3_In superlattice.

To understand the formation of ultrafine Pt_3_In clusters, the morphology, and physical structure of samples obtained from different treating temperatures were carefully probed and compared using STEM imaging. To determine the dominant growth mechanism, we analyzed the particle sizes under thermal annealing at 400, 550, 700, and 800 °C for 2 h. **Figure** **3** shows STEM images of Pt_3_In/C under different annealing temperatures. It is obvious that, as the temperature is raised, the mean diameter of the particles become larger. After 400 °C annealing for 2 h, a monodispersed cluster size of 1.0 ± 0.6 nm was obtained (Figure 3a). When annealing at 550 and 700 °C, the cluster size increased to 1.3 ± 0.7 and 1.9 ± 0.8 nm (Figure 3b,c). However, when the annealing temperature increased to 800 °C, the cluster size increased to 3.1 nm with a broader distribution, implying the occurrence of Ostwald ripening or coalescence process at very high temperature (Figure 3d).^25^ Figure 3e illustrates the atomic structure of particle of Pt_3_In/C‐T400, which shows no ordering is present after annealing at 400 °C. The diffraction pattern in Figure 3f also lacks the superlattice spots that are consistent with the atomic scale imaging in Figure 3e. By contrast, the atomic scale Z‐contrast image in Figure 3g and diffraction pattern in Figure 3h presents a clearly signature of ordered Pt_3_In structure by annealing at 700 °C. The dashed green circles in Figure 3h labels the diffraction spots corresponding to the superlattice spots of ordered Pt_3_In superlattice. Coupling with the XRD results in Figure 1c, the conclusion can be drawn that annealing at 700 °C for 2 h is sufficient to result in the formation of ultrasmall ordered Pt_3_In clusters with a narrow size distribution. The Pt‐In system is able to maintain a much smaller size without the aid of coating agent.^26^ By contrast, it has been found CdSe nanoparticles become disordered and fluxional below 2 nm.^27^ The ordered Pt_3_In structure possesses lower Gibbs energy, as shown by our ab initio molecular dynamics simulations in Figure S7 of the Supporting Information, which results in an ultrasmall size of ordered Pt_3_In nanoparticles.

**Figure 4:**
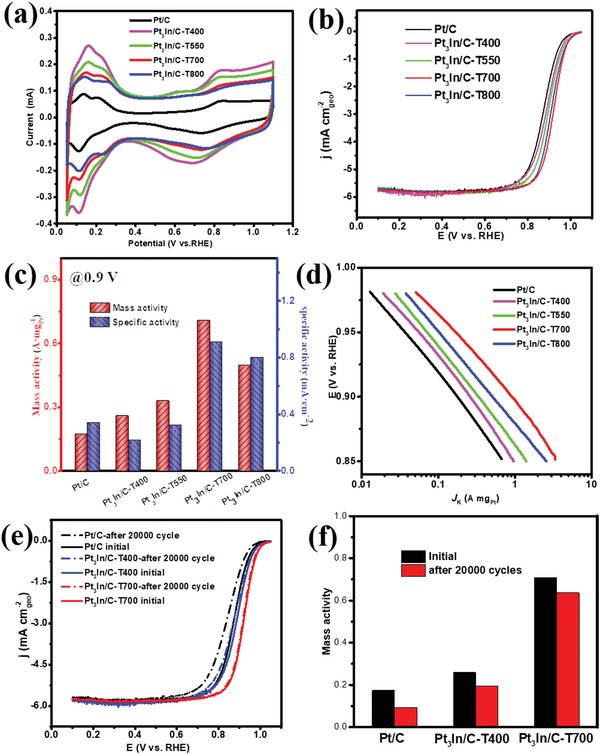
Electrocatalytic performance of different catalysts. a) CV curves recorded in N_2_‐saturated 0.1 m HClO_4_ solutions at room temperature with a sweep rate of 50 mV s^−1^. b) Positive‐going polarization curves recorded in O_2_‐saturated 0.1 m HClO_4_ solutions with a sweep rate of 10 mV s^−1^ and a rotation rate of 1600 rpm. c) Mass and specific activity at 0.9 V and d) Tafel curves. e) LSV curves before and after duration test. f) Mass activity before and after duration test.

The electrochemical behavior of the as‐prepared Pt_3_In/C catalyst for ORR was first investigated in a N_2_‐saturated 0.1 m HClO_4_ solution at a sweeping rate of 50 mV s^−1^ at room temperature. For comparison, commercial Pt/C was also examined under the same conditions. **Figure** **4**a displays the CV curves of the catalysts in N_2_‐saturated 0.1 m HClO_4_ electrolyte. The oxidation peaks at the high potential region can be assigned to the formation of adsorbed hydroxyl species on the Pt surface and the reduction peaks at the reverse scans are ascribed to the reduction of the Pt oxide. Obviously, the onset potential of the formation of absorbed hydroxyl species on Pt_3_In catalyst is significantly more positive than that of Pt/C catalysts, as well as for the reduction of Pt oxide in the backward scans. The adsorption of hydroxyl on Pt blocks active sites and inhibits the reaction of O_2_ molecules. The CV results (Table S3, Supporting Information) suggest that the formation of hydroxyl species is significantly suppressed and their desorption from the surfaces is accelerated on Pt_3_In. Thus, the more rapid recovery of the catalytic active sites for O_2_ adsorption promotes ORR on Pt‐In. The electrochemically active surface area (ECSA) of the catalysts could be calculated from the Coulombic charge involved during the hydrogen adsorption/desorption process using the expression

(1) $$\mathrm{ECSA}=Q/0.21\;\mathrm{mC}\;\;{\mathrm{cm}}^{-2}\times {\mathrm{Pt}}_{m}$$

where *Q* (mC) and Pt_m_ are the charges for hydrogen adsorption/desorption and the loading of Pt on the electrode, respectively, while 0.21 mC cm^−2^ is the electrical charge associated with monolayer adsorption of hydrogen on Pt.

The polarization curves of various electrocatalysts on a rotating‐disk electrode are obtained in an O_2_‐saturated 0.1 m HClO_4_ solution. Before the ORR activity testing, the electrode was cycling the potential between 0.05 and 1.05 V at a scanning rate of 100 mV s^−1^ for 100 cycles to obtain a clean Pt surface. Figure 4b shows that all catalysts have similar diffusion‐limited currents at higher overpotentials under the same conditions, implying they have a similar electron transfer numbers (*n*). At lower overpotentials (0.8 to 1.0 V), the current approaches the mixed kinetic and diffusion regime. In this region, the half‐wave potential (*E*_1/2_) of the polarization curve is often used to evaluate the catalytic activity of an electrocatalyst. The *E*_1_ *_/_*_2_ of the Pt_3_In/C‐T700 catalyst is 0.920 V (vs reversible hydrogen electrode (RHE)), which is 12, 25, 40, and 50 mV more positive than that of Pt_3_In/C‐T800 (0.908 V vs RHE), Pt_3_In/C‐T550 (0.895 V vs RHE), Pt_3_In/C‐T400 (0.880 V vs RHE) and the commercial Pt/C catalyst (0.870 V vs RHE), respectively. The electron transfer number (*n*) was calculated to be ≈4 at 0.8–0.9 V from the slopes of Koutecky–Levich plots from rotating‐disk voltammograms (Figure S12a,b, Supporting Information), similar to the commercial Pt/C catalyst measured in the same electrolyte (Figure S12c,d, Supporting Information). These results show that the more highly ordered Pt_3_In catalysts exhibit marked activity improvements over less‐ordered Pt_3_In and Pt/C catalysts. The superior ORR activity of Pt_3_In/C‐T700 over Pt_3_In/C‐T800 can be attributed to smaller cluster size of the former, resulting in higher Pt utilization efficiency.

For a better evaluation of the catalytic activities of the catalysts for ORR, the mass activity and specific activity at 0.9 V were calculated from the kinetic current (*I*_k_), after normalization to the loading amount and ECSA of Pt on the electrode, respectively (Figure 4b). The *I*_k_ could be calculated from the Koutecky–Levich equation based on the ORR polarization curves.^28^ The Pt_3_In/C‐T700 catalyst exhibits an outstanding mass activity of 0.71 mA mg^−1^_Pt_ at 0.9 V (vs RHE), which is almost 2.2, 2.8, and 4.2 times larger than that of Pt_3_In/C‐T550 (0.33 mA mg^−1^_Pt_), Pt_3_In/C‐T400 (0.25 mA mg^−1^_Pt_), and commercial Pt/C catalyst (0.17 mA mg^−1^_Pt_), respectively (Figure 4c). The specific activity of Pt_3_In/C‐T700 catalyst at 0.9 V is calculated to be 0.91 mA cm^−2^_Pt_, which is close to Pt_3_In/C‐T800 (0.8 mA cm^−2^_Pt_) but shows 2.8, 4.3, and 2.7 times higher than that of Pt_3_In/C‐T550 (0.32 mA cm^−2^_Pt_), Pt_3_In/C‐T400 (0.21 mA cm^−2^_Pt_), and commercial Pt/C catalyst (0.34 mA cm^−2^_Pt_), respectively. The Pt_3_In/C‐T700 exhibits a significantly enhanced ORR electrocatalytic activity by comparison to Pt_3_In clusters that are treated at lower temperatures and to commercial Pt/C catalysts, which is consistent with results presented by the CV curves discussed above.

Particle aggregation and dissolution usually occurs during harsh electrochemical‐reaction conditions, especially for small clusters with high surface energy, thus resulting in degradation of activity. To evaluate the electrochemical durability of the ordered Pt_3_In clusters, we performed an accelerated durability test (ADT) by cycling the potential between 0.6 and 1.1 V for 20 000 cycles in an O_2_‐saturated 0.1 m HClO_4_ at a scan rate of 100 mV s^−1^. The CV curves in Figure S15 of the Supporting Information show Pt_3_In‐700 clusters loss 2.1% of the ECSA, while commercial Pt/C lost its 38.8% ECSA under the same conditions. The ORR polarization curves in Figure 4e show that the Pt_3_In‐700 clusters catalyst had no substantial change in diffusion‐limited current and largely retained its ORR activity, which exhibited a negative shift in *E*_1/2_ for 3 mV after ADT test. For commercial Pt/C catalysts, the negative shifts in *E*_1/2_ are 18 mV (Figure S14, Supporting Information). Meanwhile, STEM images in Figure S16 of the Supporting Information show that Pt nanoparticles in commercial Pt/C catalysts and disordered Pt_3_In/C‐T400 suffer from obvious aggregation after the ADT test. In comparison, the ordered Pt_3_In/C‐T700 clusters showed no obvious aggregation after the ADT test (Figure S17, Supporting Information). The results indicated that the Pt_3_In‐700 cluster catalysts possess higher stability during the electrochemistry conditions in comparison to commercial Pt/C catalysts in spite of the sub‐3 nm size.

**Figure 5:**
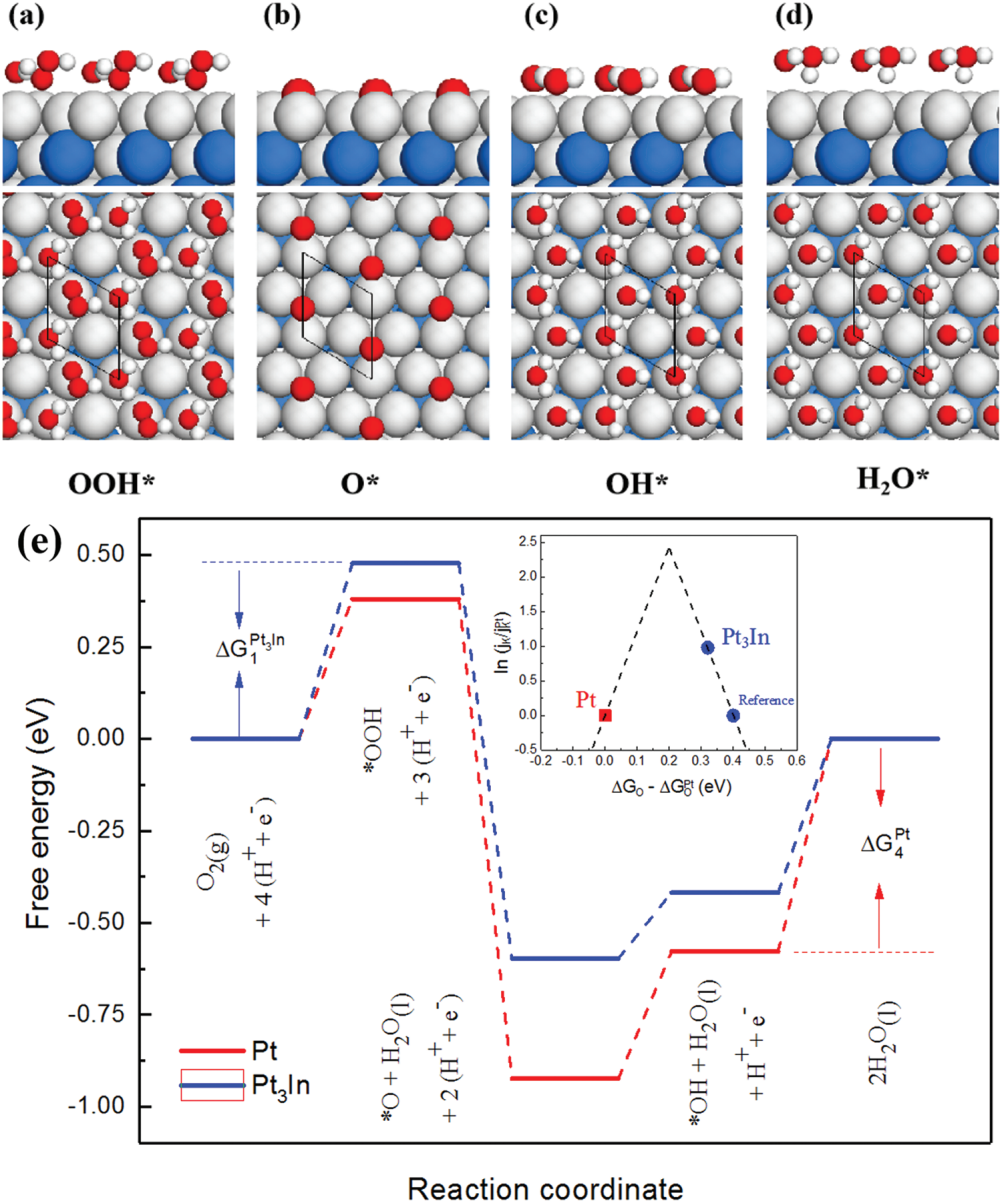
a–d) ORR intermediates adsorb at Pt sites on the Pt_3_In‐$\sqrt{3}\times \sqrt{3}R30$ supercell with an In atom in the second layer. The free energies of OOH* and OH* are calculated by including the effect of solvation. Blue, gray, red, and white spheres represent indium, platinum, oxygen, and hydrogen, respectively. e) The free energies of intermediates along the reaction pathway for ORR on Pt(111) (red line) and Pt_3_In(111) (blue line) surfaces are calculated at the equilibrium potentials (U = 1.23 V vs RHE). The stability of the intermediates is corrected for the effect of a water bilayer. The formation of OOH* and the desorption of OH* determine the over potentials of Pt_3_In(111) and Pt(111), respectively. The inset shows the volcano plot by including Pt and Pt_3_In. *j*_k_ is the current density in the experiment and Δ*G*_O_ is the calculated oxygen binding energy.

By using DFT calculations, the reaction mechanism and reaction network of ORR processes were investigated on both ordered Pt_3_In (111) and Pt (111) surfaces (Figure S1, Supporting Information). Based on the calculated free energies in Table S1 of the Supporting Information, the free energy diagram for the ORR on Pt_3_In (111) and Pt (111) surfaces at equilibrium potential (U = 1.23 V vs RHE) is shown in **Figure** **5**e. The binding energies of ORR intermediates (OOH*, O*, and OH*) on the active sites determine the activity of Pt_3_In and Pt catalysts. According to the results of calculations, oxygen binds a bit stronger on the Pt catalyst than on Pt_3_In. Furthermore, the rate‐determining step on the Pt catalyst is the last step to remove the adsorbed OH species from the surface to form water, while for Pt_3_In catalysts the rate‐determining step is the first step to transfer electron and proton to form adsorbed OOH before the O—O bond is broken. The values of Δ*G*_1_ and Δ*G*_4_, respectively, for Pt_3_In and Pt catalysts could be used as a measure of the reaction rates. The reaction‐step rate increases as the value of Δ*G* decreases. Nørskov and co‐workers reported that the ORR activity of a catalyst would be better than Pt if its surface binds O 0.00–0.40 eV more weakly than Pt, and the optimum of the volcano plot is around 0.20 eV weaker than that of Pt.^29^, ^30^ The calculated oxygen binding energy on the top of Pt_3_In(111) is 0.33 eV weaker than that on pure Pt(111), indicating that the presence of In atoms on Pt modifies the electronic structure of the Pt surface and weakens the oxygen binding. Therefore, the Pt_3_In (111) surface is a better ORR catalyst compared with Pt(111), i.e., the theoretical results are consistent with the experimental conclusions.

## 3 Conclusion

In conclusion, sub‐3‐nm intermetallic Pt_3_In clusters have been synthesized via an economic and scalable protocol and applied as highly efficient electrocatalysts in ORR for large‐scale oxygen transformation. These ordered ultrasmall Pt_3_In cluster catalysts show significantly better specific activity and mass activity than their disordered counterparts and commercial Pt/C catalysts and excellent durability in ORR. This study not only succeeds in fabricating an active and durable acid‐environment ORR catalyst but also demonstrates a general and effective approach to obtain ultrasmall ordered nanoalloys for heterogeneous catalytic applications.

## 4 Experimental Section

*Synthesis*: An impregnation method was used for the large‐scale synthesis of intermetallic Pt_3_In clusters. Instead of using well‐defined graphene or carbon nanotubes, commercially available KJ carbon ECP600JD was used as an inexpensive support. First, the KJ carbon support was treated with 1 m HNO_3_ at 160 °C for 6 h to activate the surface, i.e., to generate more defects that are needed in later process steps. The activated carbon was then dispersed in ethanol (typically, 76 mg carbon in 30 mL ethanol). Meanwhile, 10.0 mg of InCl_3_·4H_2_O and 53.3 mg of H_2_PtCl_6_·6H_2_O were dissolved in 10 mL of ethanol. Subsequently, the mixed solution was added dropwise into the presonicated suspension of KJ carbon. After ultrasonic mixing for 2 h, the suspension was dried by vacuum rotary evaporation. Then, the resulting black powder was heated in a tube furnace at 400, 550, 700, and 800 °C under 10% H_2_/Ar flowing for 2 h with a heating rate of 5 °C per minute. Finally, the powder was cooled to room temperature in a N_2_ atmosphere and Pt_3_In/C was harvested. To scale up the precursor and carbon, large‐scale Pt_3_In/C was obtained. The as‐obtained powder was further treated with 0.1 m HClO_4_ at 70 °C for 12 h to remove excess indium atoms on the surface. The samples obtained under different temperatures were labeled as Pt_3_In/C‐T400, Pt_3_In/C‐T550, Pt_3_In/C‐T700, and Pt_3_In/C‐T800.

*Characterization*: Crystallographic information for the electrocatalysts was obtained by powder XRD using a Shimadzu XRD‐7000. HAADF‐STEM images and energy‐dispersive X‐ray spectrometry (EDX) images were obtained in an aberration‐corrected Titan Themis with an operating voltage of 300 kV. X‐ray photoemission analysis were carried out on a PHI 5000 VersaProbe II spectrometer using a monochromatic Al K(alpha) X‐ray source.

The electrochemical catalysis experiments were performed with a CHI 760E in a three‐electrode cell. A RHE filled with H_2_‐saturated 0.1 m HClO_4_ was used as the reference electrode and a Pt mesh electrode (1.5*1.5 cm^2^) was used as counter electrode. 1 mg carbon‐supported catalyst was mixed with 990 µL isopropanol and 10 µL Nafion (5%) and sonicated for 30 min. To prepare the working electrode, catalyst ink containing 3 µg of Pt was cast onto a glass carbon rotating disk electrode (5 mm in diameter) and dried under ambient conditions. The Pt loading for all catalysts is about 15.3 µg cm^−2^_geo_ on the working electrode. Cyclic voltammetry curves were conducted in N_2_‐saturated 0.1 m HClO_4_ at a scanning rate of 50 mV s^−1^. The ORR polarization curves were recorded in O_2_‐saturated 0.1 m HClO_4_ at a scanning rate of 10 mV s^−1^ with iR drop correction. The stability of the catalysts was evaluated by potential sweeps between 0.6 and 1.1 V for 20 000 cycles.

*DFT Calculation*: All calculations were carried out using the Vienna Ab Initio Simulation Package^31^, ^32^ based on DFT.^33^, ^34^ The Perdew–Burke–Ernzerhof functional in the framework of the generalized gradient approximation was adopted.^35^ The interactions between ions and electrons were described by the projector augmented wave method.^36^ A cut‐off energy of 500 eV was applied for the plane‐wave basis set, and a k‐space mesh of 10 × 10 × 1 was used. To simulate the adsorption behavior of intermediates on the surface, Pt_3_In (111) and Pt (111) slabs containing four close‐packed layers were built, in which the bottom two layers were fixed. The vacuum layer was larger than 13 Å in order to guarantee negligible interactions between the periodically repeated slabs. All structures were fully relaxed until the forces acting on each atom were smaller than 0.02 eV Å^−1^. More theoretical details are included in the Supporting Information.

## Conflict of Interest

The authors declare no conflict of interest.

## Supporting information

Supporting Information
