# Supplementary material for: Sub‐3 nm Intermetallic Ordered Pt3In Clusters for Oxygen Reduction Reaction
Source: Adv Sci (Weinh). 2019 Nov 18;7(2):1901279. doi: 10.1002/advs.201901279 (PMC6974934; doi:10.1002/advs.201901279)
Supplement: Supplementary file 1 — Supporting Information [file ADVS-7-1901279-s001.pdf]

Copyright WILEY-VCH Verlag GmbH & Co. KGaA, 69469 Weinheim, Germany, 2019.

ADVANCED SCIENCE
Open Access

Supporting Information

for *Adv. Sci.,* DOI: 10.1002/advs.201901279

Sub-3 nm Intermetallic Ordered Pt_3In Clusters for Oxygen Reduction Reaction

Qi Wang, Zhi Liang Zhao, Zhe Zhang, Tianli Feng, Ruyi Zhong, Hu Xu, Sokrates T. Pantelides,* and Meng Gu**

Supporting Information

Sub-3 nm Intermetallic ordered Pt_3In Clusters for Oxygen Reduction Reaction

Qi Wang[1,3‡], Zhi Liang Zhao[1‡], Zhe Zhang[2‡], Tianli Feng[4], Ruyi Zhong[1], Hu Xu[2], Sokrates T. Pantelides[4]*, Meng Gu[1]**

Qi Wang, Zhi Liang Zhao and Zhe Zhang contributed equally to this paper

[1]Department of Materials Science and Engineering, and [2]Department of Physics, Southern University of Science and Technology, Shenzhen 518055, China
[3] Department of Materials Science & Engineering, University of Science and Technology of China, Hefei 230026, PR China
[4]Department of Physics and Astronomy and Department of Electrical Engineering and Computer Science, Vanderbilt University, Nashville, TN 37235, USA
E-mail: gum@sustech.edu.cn

Keywords: Intermetallic ordered, Pt_3In, sub-3 nm clusters, large-scale, oxygen reduction reaction

Table of content

1. Experimental Details 2
2. Computational details 2
3. Microscopy and spectroscopy characterization 8
4. Structure and Performance of Pt_2In/C-T700 and PtIn/C-T700 12
5. Electrocatalytic performance 15
6. Structure characterization of catalyst after ADT test 19
7. Reference 21

1. Experimental Details

Materials

Hydrogen hexachloroplatinate(IV) ($H_2PtCl_6·6H_2O$, 99.995%) and Indium chloride ($InCl_3$· $4H_2O$) were purchased from Aladdin. Nafion (5 wt %) were purchased from Sigma-Aldrich. Ketjenblack EC-600JD carbon was supplied by Normal KB (Lion Corporation, Japan, EC - 600JD). Concentrated nitric acid (HNO_3, 65%) and perchloric acid ($HClO_4$, 70%) were purchased from Sinopharm Chemical Reagent Co. Ltd. (Shanghai, China). All reagents were used as received without further purification.

Characterization

The STEM images were obtained using a double Cs-corrected Themis Z S/TEM operated at an accelerating voltage of 300 kV (80-300 kV, 0.08 nm spatial resolution in STEM). Samples were sonicated and dispersed in alcohol. One drop of the solution was added onto a Lacey Carbon copper grid and dried on a hot plate at 70 °C. The phase of the as-prepared products was identified by XRD with Cu Kα radiation (λ = 1.5418 Å). The XPS analysis was carried out on a PHI 5000 VersaProbe II spectrometer using monochromatic Al K(alpha) X-ray source.

2. Computational details

2.1 Calculated the free energies for different intermediates on different surfaces

The binding energy of the ORR intermediates on Pt and Pt_3In was calculated using a $\sqrt{3}\times\sqrt{3}R30^{\circ}$ surface unit cell. The Gibbs free energy of the adsorbates is calculated by

$G=E_{DFT}+E_{ZPE}-TS$. The E_{DFT} is the calculated energy. E_{ZPE} and TS respectively represent the zero point energy correction from vibrational frequencies and entropy contribution.[[1]] In order to minimize the variations of the absolute Gibbs free energy for adsorbates on Pt and Pt_3In, the E_{ZPE} is assumed not change with the surface composition as described in a previous publication.[[2]] Figure S1 shows the most stable surface structures of Pt (111). The effects of solvation for adsorbed OOH and OH at the water–solid interface are considered in the calculations. The total coverage is 2/3 monolayer for OOH/H_2O and OH/H_2O and their adsorption energies are calculated by

$$\Delta E_{OOH} = E_{OOH/H_2O} + \frac{3}{2}E_{H_2} - 3E_{H_2O} - E_{slab} - \Delta E_{H_2O}$$

$$\Delta E_{OH} = E_{OH/H_2O} + \frac{1}{2}E_{H_2} - 2E_{H_2O} - E_{slab} - \Delta E_{H_2O}$$

where E_{OOH/H_2O} and E_{OH/H_2O} represent the DFT energies of the superstructures for OOH/H_2O and OH/H_2O, respectively. E_{slab} is the substrate energy. E_{H_2} and E_{H_2O} are calculated in the gas phase. ΔE_{H_2O} is the adsorption energy of single water molecule in an ordered H_2O/H_2O-$\sqrt{3}\times\sqrt{3}R30°$ unit cell.[[3]] The free energies of OOH*, O* and OH* are shown in Table S1.

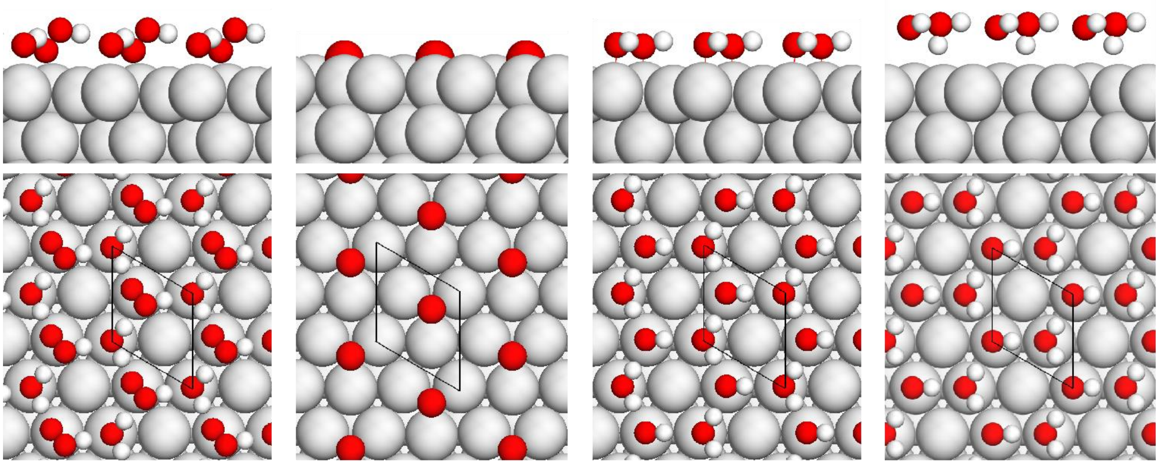

Figure S1. ORR intermediates adsorbed at Pt sites on Pt-$\sqrt{3}\times\sqrt{3}R30°$ unit cell. The free energies of OOH* and OH* have been calculated including the effect of solvation. Gray, red and white spheres represent platinum, oxygen and hydrogen, respectively.

We also build the slab models with Pt/In ratios of 1:1 and 2:1, and calculate their ORR free energy diagrams at the equilibrium potential, i.e., U=1.23 V vs RHE. For the Pt(111) surface, the potential-determining step is the last step (OH* + H^+ + e^- → * + H_2O), while it changes to the first step (* + O_2 + H^+ + e^- → OOH*) on Pt_3In(111), Pt_2In(111), and PtIn(111), as shown in Figure S2a. This is ascribed to the fact that doping of In weakens the binding energies of ORR intermediates (Table S1). The calculated overpotentials of Pt_3In(111), Pt_2In(111), and PtIn(111) are 0.48 eV, 0.50 eV, and 0.54 eV, respectively, which are smaller than that of Pt(111) (0.58 eV), indicating that doping of In enhances the ORR activity of Pt(111). Moreover, the ORR activities decrease with the increase of In ratio, i.e., Pt_3In(111) > Pt_2In(111) > PtIn(111), while they are still better than Pt(111). To verify this trend, we conduct experiments to measure the kinetic current density (j_k) of these samples. As shown in Figure S2b, the relative ORR activities of these samples are well consistent with our DFT calculations.

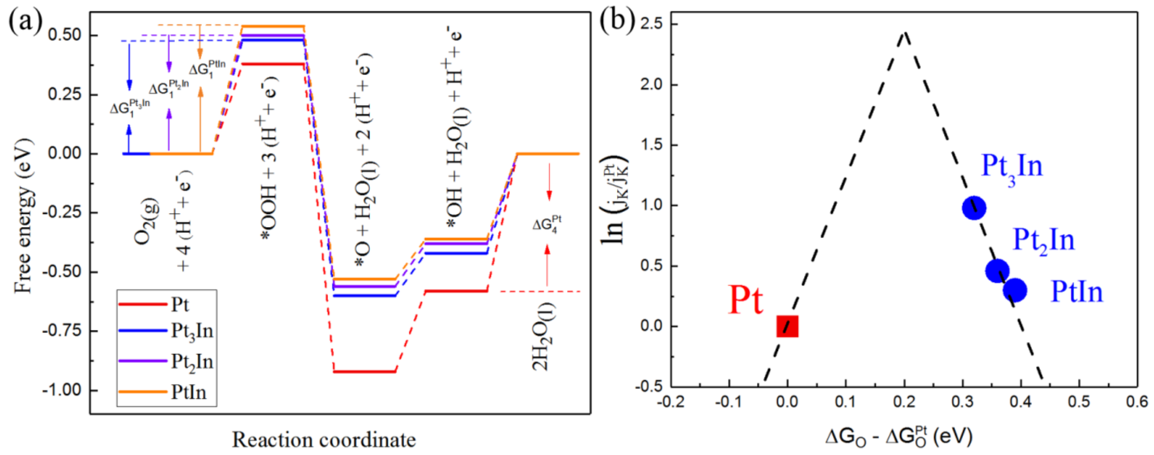

Figure S2. (a) Free energy diagrams of ORR on Pt(111) (red line), Pt_3In (111) (blue line), Pt_2In (111) (purple line), and PtIn (111) (orange line) at the equilibrium potential (U=1.23 V *vs* RHE). (b) The measured current density j_k of Pt, Pt_3In, Pt_2In, and PtIn plotted as a function of oxygen binding energy.

In this study, we consider the solvation effect for OH* and OOH* using a half-dissociated water layer network, which is regarded as the most stable structure on Pt(111) at the ORR working potentials.[[2, 4-6]] Figure S3a-b show these structures composed of 1/3 monolayer of OOH* or OH* and 1/3 monolayer of H_2O* in a $\sqrt{3}\times\sqrt{3}$ R30 supercell, in which each OOH* or OH* is hydrogen bonded with three adjacent H_2O molecules, leading to the maximum number of hydrogen bonds. In contrast to OH* and OOH* which prefer to adsorb on the top site of (111) surface, the O* are energetically favorable to occupy the hollow site, which results in the water layer floating above the O* (see Figure S3c). On the whole, we consider the solvation effect of OH*, OOH*, and O* by incorporating them in a stable hydrogen bonded network. Based on these configurations, we calculate the solvation corrections for O* is -0.02 eV, which is well consistent with the prior studies (see Figure S3d).[[1-2, 6]]
The influence of vdW interaction (DFT-D3) on the ORR on Pt(111) and Pt_3In (111) is considered. It can be seen from Figure S3e, the vdW interaction strengthens the binding energies of OOH*, O* and OH* (Table S1**)**, which alters the potential-determining step on Pt_3In(111) from the first step (* + O_2 + H^+ + e^- → *OOH) to the last step (*OH + H^+ + e^- → H_2O +*). Moreover, the overpotentials increase from 0.58 V to 0.87 V and from 0.48 V to 0.72 V on Pt(111) and Pt_3In (111), respectively, as shown in Figure S3e. Furthermore, we use HSE functional to perform the ORR simulation. The calculated overpotential of Pt_3In(111) is 0.76 V, which is 0.28 V larger than that obtained from GGA-PBE, as shown in Figure S3f.

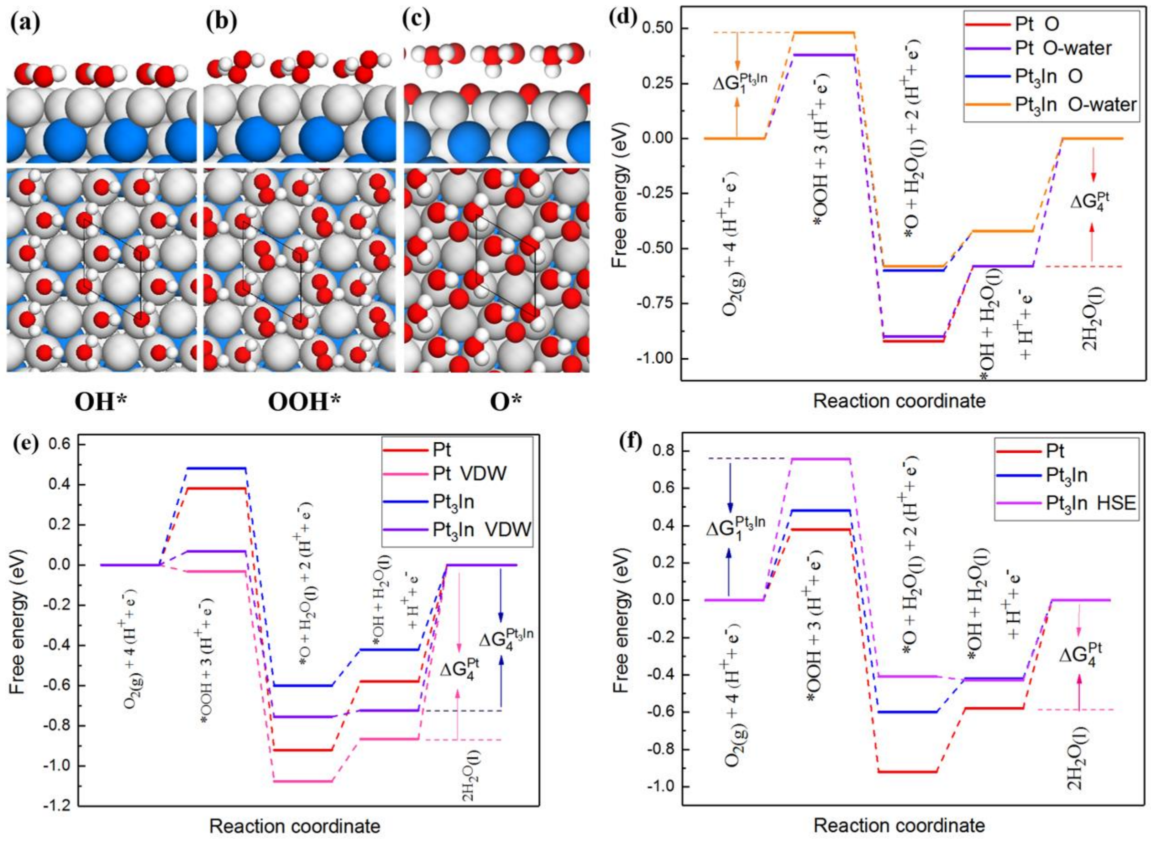

Figure S3. (a)-(c) Optimized configurations of ORR intermediates on the Pt_3In(111) surface. Pt, In, O, and H atoms are represented by gray, blue, red, and white spheres, respectively. (d) Free energy diagrams of ORR on Pt (111) and Pt_3In (111) at the potential of 1.23 V *vs* RHE with and without consideration of solvation correction to O*. (e) Free energy diagrams of ORR on Pt (111) and Pt_3In (111) at the potential of 1.23 V *vs* RHE with and without consideration of vdW interaction. (f) Free energy diagram of ORR calculated using HSE functional.

Table S1. Calculated free energies (units in eV) of ORR intermediates on the (111) surfaces of Pt, Pt_3In, Pt_2In, and PtIn at the potential of U=1.23 V *vs* RHE with PBE, PBE+D3, and HSE functionals, respectively.

Surface	ΔG(OOH*)	ΔG(O*)	ΔG(OH*)	ΔG(O*+H_2O)	$ΔG_1$	$ΔG_4$
Pt(111)	0.38	-0.92	-0.58	-0.90	—	0.58
Pt_3In(111)	0.48	-0.60	-0.42	-0.58	0.48	—
Pt (111) -vdW	-0.03	-1.08	-0.87	—	—	0.87
Pt_3In(111)-vdW	0.07	-0.76	-0.72	—	—	0.72
Pt_3In(111)-HSE	0.76	-0.41	-0.43	—	0.76	—

A periodic slab model is used to simulate the 3-nm sized Pt_3In cluster. To verify the validity of this model, we construct three Pt_3In cuboctahedral clusters of different sizes according to the method proposed in the previous studies.[[7-8]] These clusters are composed of 147, 309 and 561 atoms, respectively, as shown in Figure S4. Because the binding energy of O* (ΔG_{O*}) is the ORR activity descriptor for transition metal catalysts, we calculate the ΔG_{O*} on the (111) surfaces of these clusters to estimate their ORR activities.[[1, 6, 9]] As listed in Table S2, the ΔG_{O*} gradually increases as the cluster size increases. This trend is consistent with the prior study which indicates that the electronic finite-size effects of Pt cluster vanish when the size is beyond 1.6 nm.[[7]] Therefore, it is reasonable to use a slab model to simulate 3-nm sized Pt_3In in ORR.

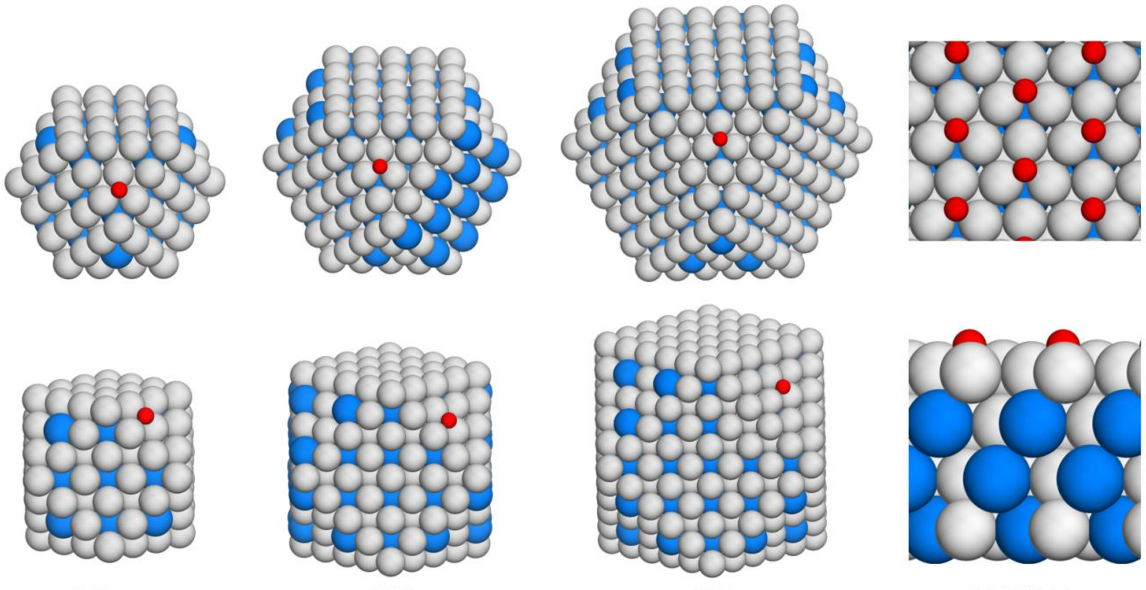

Figure S4. Top and side views of three Pt_3In cuboctahedra clusters composed of 147, 309 and 561 atoms, respectively. The oxygen atom is placed at the fcc hollow site close to the center of (111) surface of the cluster. The spheres colored in gray, blue, and red denote the Pt, In, and O atoms, respectively.

Table S2. Calculated binding energies of O* on the (111) surfaces of Pt_3In clusters with different sizes shown in Figure S4.

Pt_3In cuboctahedra clusters (atoms)	147	309	561	O(111) Fcc
ΔG_{O*} (eV)	1.546	1.621	1.743	1.863

2.2 AIMD simulations

To verify the experimental observations and find the critical size of nanoparticles that can stay crystalline at high temperatures, we carried out ab initio molecular dynamics (AIMD) simulations. We start the simulations using cubic nanoparticles with sizes 0.4*0.4*0.4, 0.6*0.6*0.6, 0.8*0.8*0.8, and 1.2*1.2*1.2 nm^3 to investigate the stability of crystalline nanoparticles, with the structures shown in Figure S4. Before AIMD simulations, we relaxed the structures until the atomic force on each atom is smaller than 0.1 eV/Å. The vacuum space in the simulation domain along each direction is set as 20 Å to make sure the nanoparticles do not interact with their images. The AIMD simulations were run at 973 K (700 C) in a canonical ensemble (NVT) for 10,000 steps. The AIMD results clearly show that nanoparticles with size smaller than 1*1*1 nm^3 quickly turn to amorphous while the one with dimensions 1.2*1.2*1.2 nm^3 keep their crystalline form. We increased the simulation time for the last case and did not observe a transformation into amorphous. Therefore, we conclude that ~1.2 nm is the critical size for InPt3 nanoparticles to stay crystalline at 973 K (700 C).

2.3 Details for DFT AIMF simulations:

The first-principles density functional theory (DFT) simulations are performed via the VASP package.[10] We used the Perdew-Burke-Ernzerhof (PBE) parameterization of the generalized gradient approximation (GGA) for exchange and correlation functionals[11] with the projector-augmented-wave method.[12] The plane-wave energy cutoff is 400 eV, and the electron k mesh is taken as the Γ point for the AIMD simulations. Each step of AIMD simulation is 0.5 fs.

3. Microscopy and spectroscopy characterization

3.1 Large-scale STEM characterization of Pt_3In/C-T700.

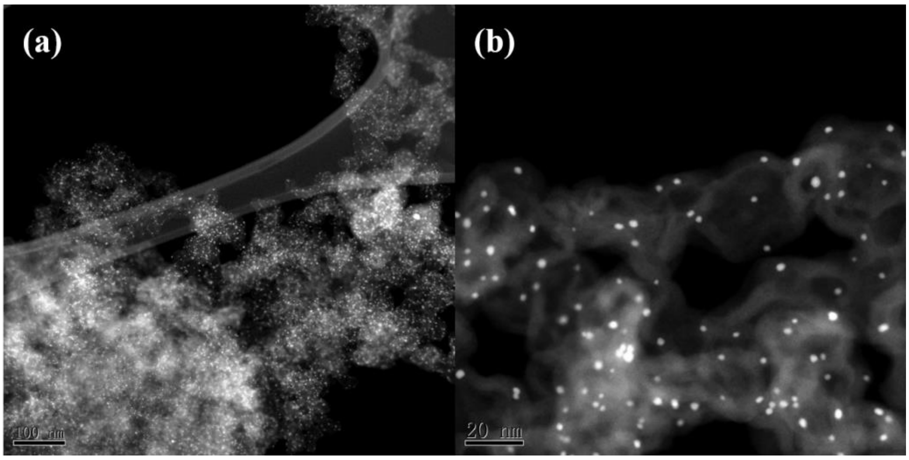

Figure S5 STEM images of Pt_3In/C-T700 in different magnifications

3.2 Large-scale STEM images showing Pt_3In/C-T400 at different magnifications

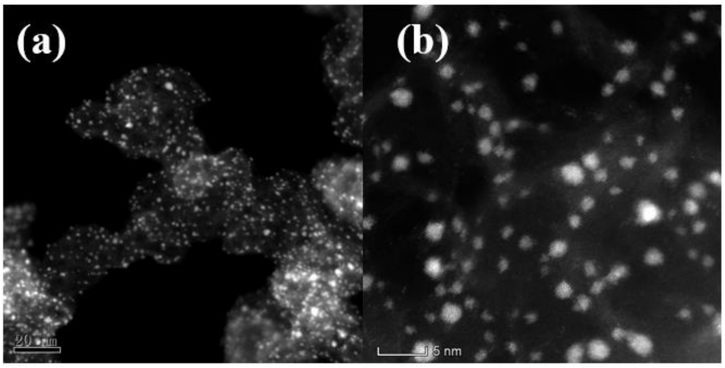

Figure S6 STEM images of Pt_3In/C-T400 in different magnifications

3.3 AIMD simulation of the stability of ordered cluster

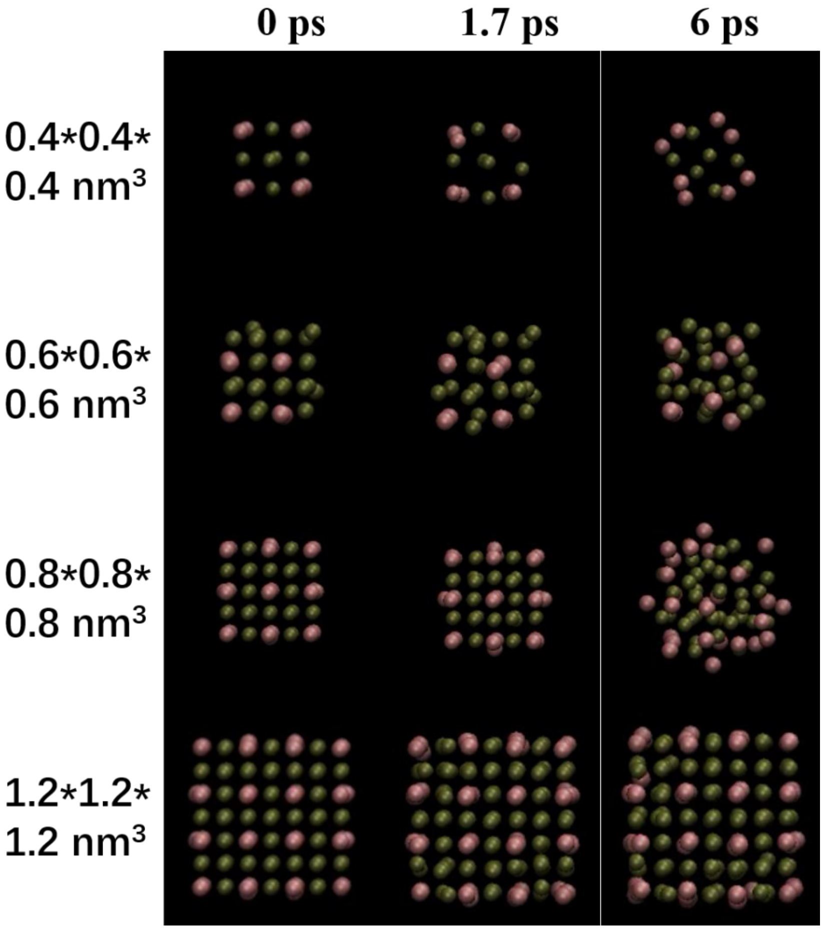

Figure S7 time-dependent images of AIMD simulation of ordered Pt_3In in different size under 700℃

3.4 TEM result of Pt_3Co prepared under same method

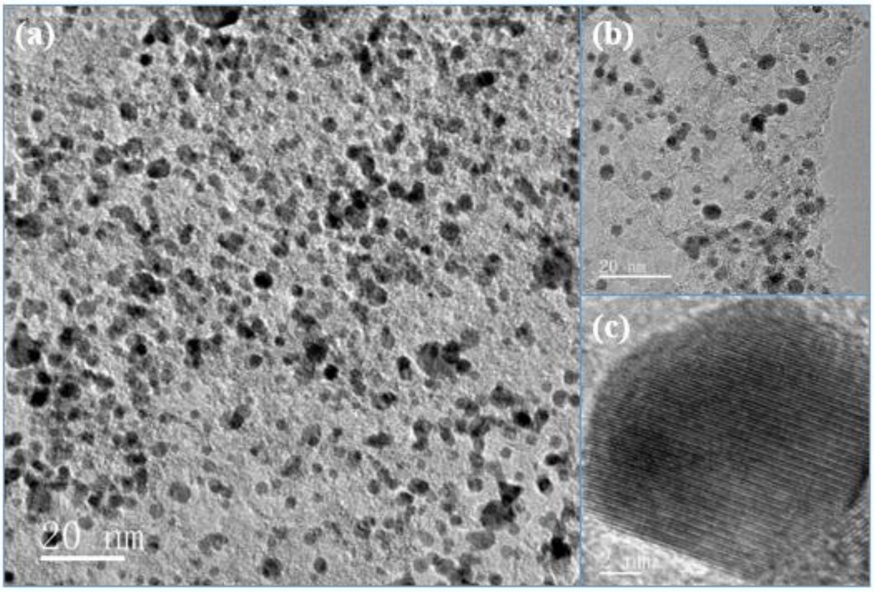

Figure S8 TEM images of ordered Pt_3Co synthesized by this general method

4. Structure and Performance of Pt_2In/C-T700 and PtIn/C-T700

To investigate the influence of In content to the ORR performance, we synthesis Pt_2In/C-T700 and PtIn/C-T700. Structure and Performance characterization are present in this chapter.

4.1 STEM images of Pt_2In/C-T700 and PtIn/C-T700

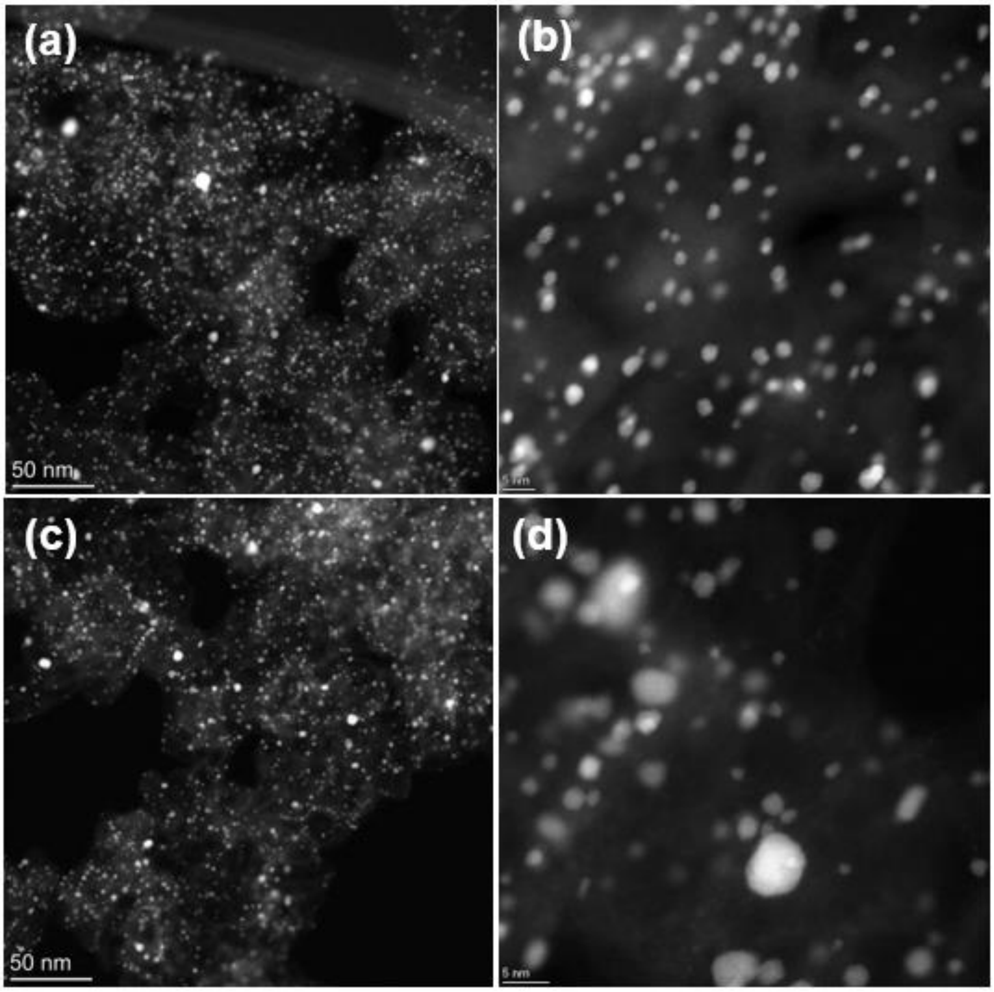

Figure S9 (a-b) STEM images of Pt_2In/C-T700 in different magnifications and (c-d) STEM images of PtIn/C-T700 in different magnifications

4.2 XRD result of Pt_2In/C-T700 and PtIn/C-T700

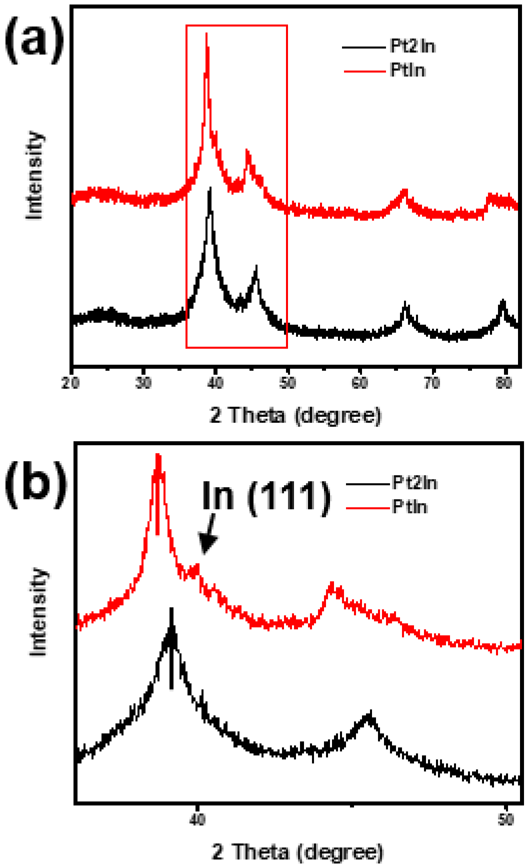

Figure S10 (a) XRD patterns of Pt_2In/C-T700 and PtIn/C-T700 and (b) the enlarged region of red region indicate the separation of excess In. The result reveal that increased In content may resulted in precipitation of In.

4.3 Electrochemical test of Pt_2In/C-T700 and PtIn/C-T700

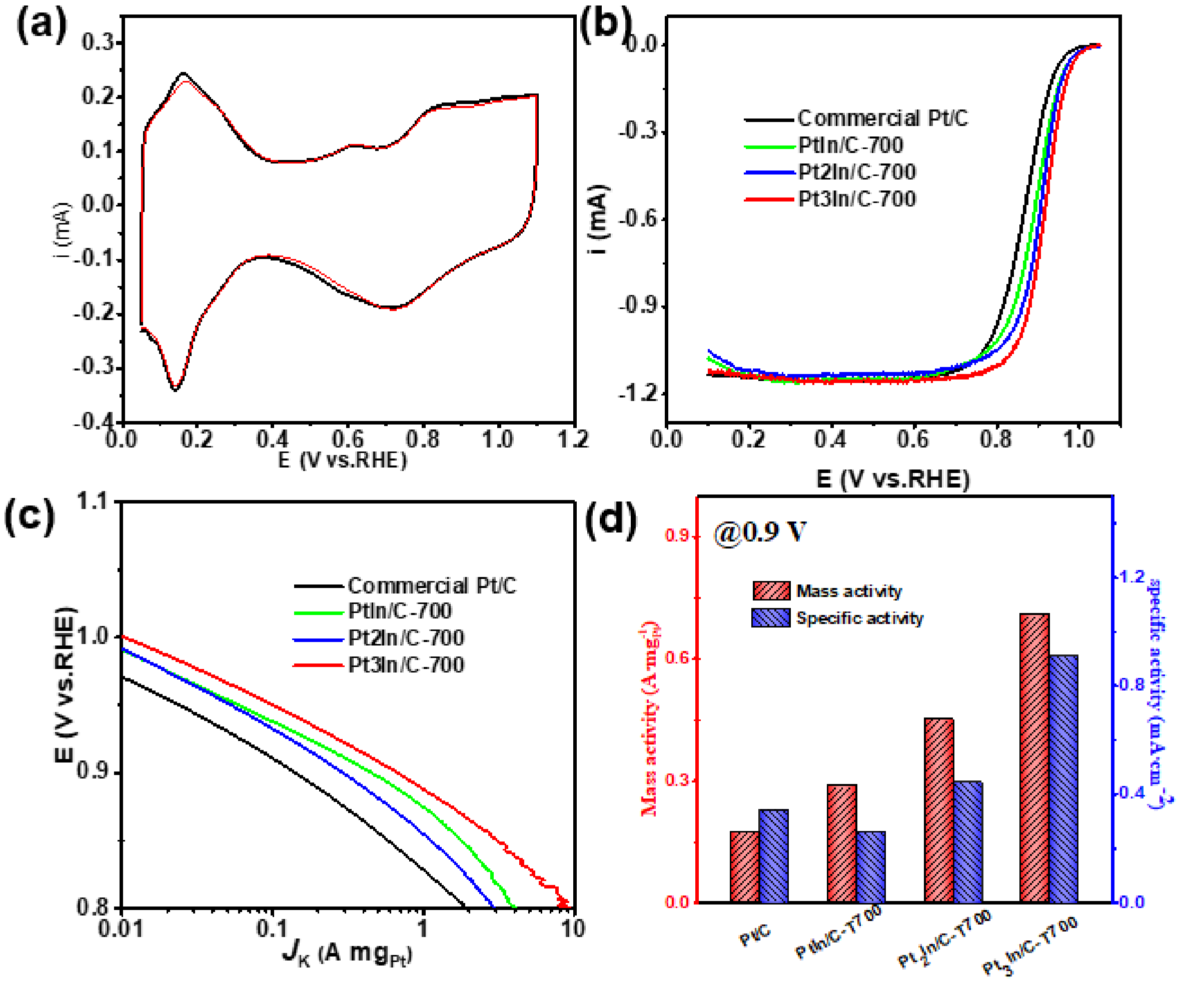

Figure S11 Electrocatalytic performance of Pt_2In/C-T700 and PtIn/C-T700. (a) CV curves recorded in N_2-saturated 0.1 M $HClO_4$ solutions at room temperature with a sweep rate of 50 mV s^{-1}. (b) Positive-going polarization curves recorded in O_2-saturated 0.1 M $HClO_4$ solutions with a sweep rate of 10 mV s^{-1} and a rotation rate of 1600 rpm. (c) Tafel curves and (d) Mass and specific activity at 0.9 V.

The $E_{1/2}$ of the PtIn/C-T700 catalyst and Pt_2In/C-T700 catalyst is 0.893 V and 0.902 (Vs RHE), respectively, which is lower than the Pt_3In/C-T700 catalyst and higher than commercial Pt/C. The mass activity of PtIn/C-T700 and Pt_2In/C-T700 are 0.286 mA mg^{-1}_{Pt} and 0.451 mA mg^{-1}_{Pt}, respectively, lower than the Pt_3In/C-T700 catalyst.

5. Electrocatalytic performance

5.1 Rotating-disk voltammograms

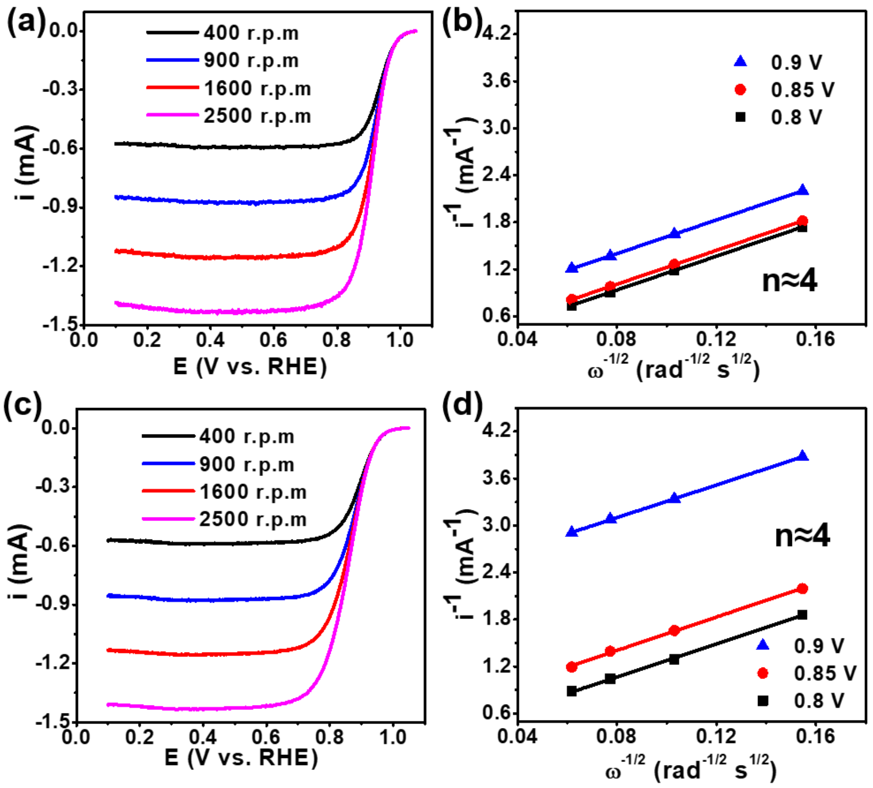

Figure S12. Rotating-disk voltammograms of (a) Pt_3In and (c) commercial Pt/C in O_2-saturated 0.1 M $HClO_4$ with a sweep rate of 5 mV s^{-1} at the different rotation rates indicated. (b) and (d) show corresponding Koutecky–Levich plots (i^{-1} versus $\omega^{-0.5}$) at different potentials.

Table S3 Summary of electrochemical performance

Sample	half-wave potential ($E_{1/2}$, V)	mass activity (mA mg^{-1}_{Pt})	ECSA (cm^2/g)
Commercial Pt/C	0.870	0.17	50.1
Pt_3In/C-T400	0.880	0.25	120.68
Pt_3In/C-T550	0.895	0.33	102.7
Pt_3In/C-T700	0.920	0.71	78.48
Pt_3In/C-T800	0.908	0.49	61.8

5.3 Electrocatalytic performance of Pt_3Co/C catalyst

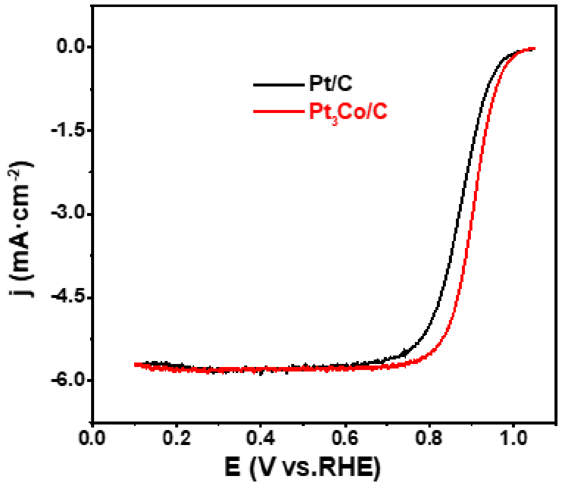

Figure S13 LSV of ordered Pt_3Co/C catalyst

5.4. Stability test of Pt_3In/C and commercial Pt/C

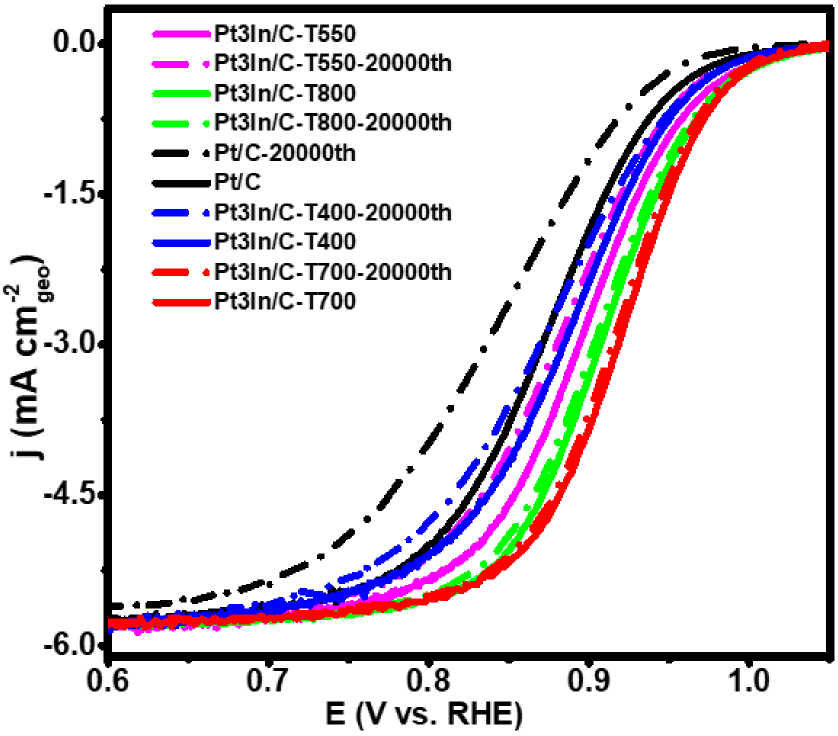

Figure S14 LSV curves before and after duration test of all samples.

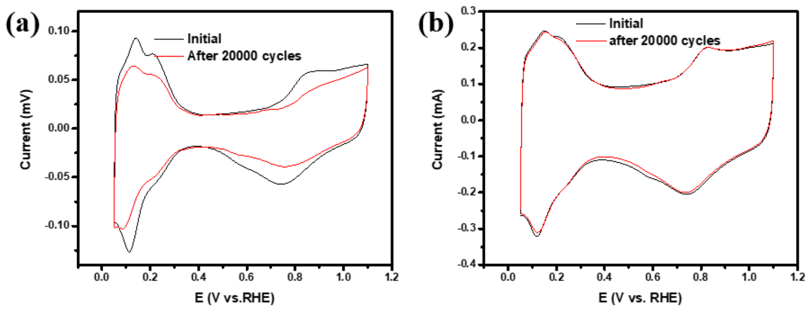

Figure S15 CV curves of (a) Pt/C and (b) Pt_3In/C-T700 before and after 20,000 cycles

6. Structure characterization of catalyst after ADT test

6.1 TEM images of commercial Pt/C and dis-ordered Pt_3In/C catalyst

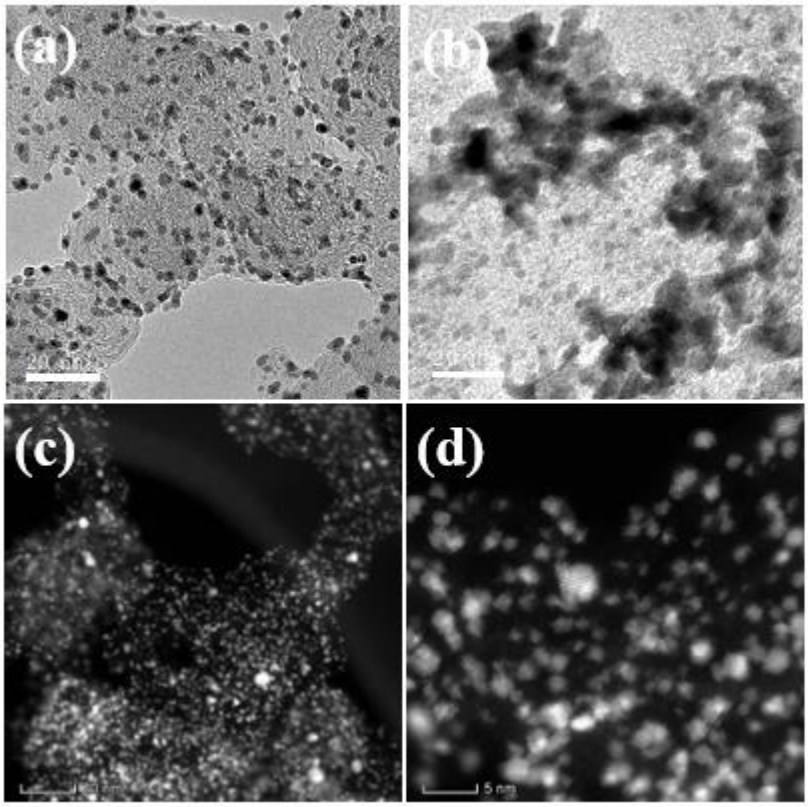

Figure S16 (a-b) TEM images of Pt/C before (a) and after (b) duration test. (c-d) STEM images of disordered Pt_3In/C-T400 before (c) and after (d) duration test.

6.2 Structure of Pt_3In-700/C after ADT test

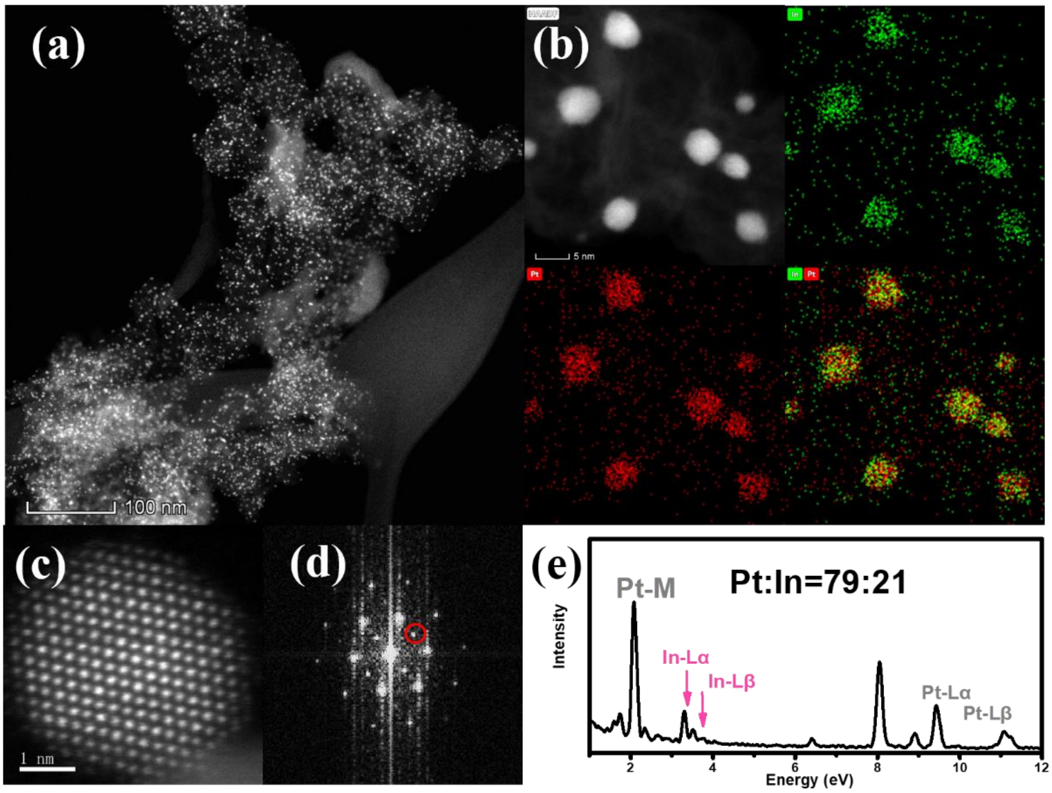

Figure S17 (a) STEM images and (b) EDS-mapping of ordered Pt_3In/C-T700 after duration test. (c) and corresponding (d)FFT patterns indicates the maintenance of ordered structure. (e) EDS-spectrum after duration test.

7. Reference

[1] J. K. Norskov, J. Rossmeisl, A. Logadottir, L. Lindqvist, J. R. Kitchin, T. Bligaard, H. Jonsson, *J Phys Chem B* **2004**, *108*, 17886-17892.

[2] J. Greeley, I. E. Stephens, A. S. Bondarenko, T. P. Johansson, H. A. Hansen, T. F. Jaramillo, J. Rossmeisl, I. Chorkendorff, J. K. Norskov, *Nat Chem* **2009**, *1*, 552-556.

[3] S. Meng, E. G. Wang, S. Gao, *Physical Review B* **2004**, *69*.

[4] V. Tripković, E. Skúlason, S. Siahrostami, J. K. Nørskov, J. Rossmeisl, *Electrochimica Acta* **2010**, *55*, 7975-7981.

[5] J. Rossmeisl, A. Logadottir, J. K. Nørskov, *Chemical Physics* **2005**, *319*, 178-184.

[6] J. Rossmeisl, J. K. Norskov, C. D. Taylor, M. J. Janik, M. Neurock, *J Phys Chem B* **2006**, *110*, 21833-21839.

[7] L. Li, A. H. Larsen, N. A. Romero, V. A. Morozov, C. Glinsvad, F. Abild-Pedersen, J. Greeley, K. W. Jacobsen, J. K. Norskov, *J Phys Chem Lett* **2013**, *4*, 222-226.

[8] J. Kleis, J. Greeley, N. A. Romero, V. A. Morozov, H. Falsig, A. H. Larsen, J. Lu, J. J. Mortensen, M. Dułak, K. S. Thygesen, J. K. Nørskov, K. W. Jacobsen, *Catalysis Letters* **2011**, *141*, 1067-1071.

[9] Z. W. Seh, J. Kibsgaard, C. F. Dickens, I. Chorkendorff, J. K. Norskov, T. F. Jaramillo, *Science* **2017**, *355*.

[10] G. Kresse, J. J. P. R. B. Hafner, **1993**, *47*, 558.

[11] J. P. Perdew, K. Burke, M. J. P. r. l. Ernzerhof, **1996**, *77*, 3865.

[12] G. Kresse, D. J. P. R. B. Joubert, **1999**, *59*, 1758.
